# Oncology During the COVID-19 Pandemic: a Lockdown Perspective

**DOI:** 10.1007/s11912-022-01301-4

**Published:** 2022-05-27

**Authors:** Danielle Boniface, Gonzalo Tapia-Rico

**Affiliations:** 1 School of Medicine, University of Adelaide, Adelaide, South Australia Australia; 2 Icon Cancer Centre Adelaide, Kurralta Park, South Australia 5037 Australia

**Keywords:** COVID-19, SARS-Cov-2, Pandemic, Oncology, Cancer

## Abstract

**Purpose for Review:**

This perspective piece aims to understand the impacts of the COVID-19 pandemic on the field of oncology, exploring the factors provoking a fall in cancer diagnostic rates, interruption of cancer screening programmes, disruption of oncological treatment and adjuvant care, and the necessary adaption oncological practice has undergone (and will be required to undergo) post-pandemic, including the shift to digital consultations.

**Recent Findings:**

During the COVID-19 pandemic, the field of oncological research has faced significant challenges. Yet, innovation has prevailed with new developments being made across the globe. Looking to the future of oncology, this piece will also suggest potential solutions to overcome the late-stage ramifications of the COVID-19 pandemic.

**Summary:**

The COVID-19 pandemic has triggered a global health crisis, the ramifications of which have reached every corner of the world and overwhelmed already overburdened healthcare systems. However, we are still yet to see the full domino effect of the pandemic as it continues to reveal and exacerbate pre-existing weaknesses in healthcare systems across the world.

## Introduction

As the COVID-19 pandemic rages on, it is time to reflect on the toll it has taken on the field of oncology. The ‘unprecedented’ impacts have been wide-sweeping, with no corner of the globe spared. Along with the biophysical cost of ill-health and mortality, the pandemic bought with it major sociocultural upheaval. Research on the sociology of pandemics suggests that these enduring consequences will affect oncological practice inequitably between groups of different socioeconomic status (SES) [1, 2•]. This perspective draws on sources from Europe, North America and Australia to shed light on trans-national concerns over falls in cancer diagnosis and active treatment rates. The nature of practicing oncology has substantially shifted during the pandemic, and we will explore these adaptions from both a practitioner and patient perspective. Turning to the challenges and triumphs of medical research, this perspective will reveal how studies have been halted in some areas, while innovation is emerging in others. Finally, we examine the legacy effects of COVID-19 as we move towards a post-pandemic era, with a focus on and what this may mean for the future of oncology and challenges we are yet to encounter as we adjust to our ‘new-normal’.

## Fall in Diagnostic Rates of Cancers

### The Statistics

The pandemic has sidelined the public’s attention to cancer, with ‘huge numbers of missed diagnoses’ occurring globally [3••]. In early 2020 Europe, the number of cancer diagnoses fell by 30–40% in the Netherlands and Belgium, and by a monumental 90% in Kyrgyzstan [4]. Italy (who suffered a particularly heavy burden of COVID-19 during the first wave of the pandemic) experienced a 39% decrease in cancer diagnoses during 2020, compared to average rates from previous years [5]. During the UK’s first lockdown of March 2020, the number of referrals to oncologists of suspected cancers reduced by 350,000, which is a 76% decrease compared to the same period in 2019 [3••].

Worryingly, some of the cancers which suffered the greatest falls in diagnosis were those which typically follow a very rapid and catastrophic course with poor prognoses when diagnosed at an advanced stage. Of all cancers included in this study, the most concerning change was the 62% decrease in diagnostic rates of colorectal cancer (CRC), as even short delays in initiating treatment of CRC have been shown to significantly increase patients’ risk of death [6]. Another significantly affected cancer was skin cancer as the Dutch National Cancer Registry [7] recorded a 60% drop in diagnosis of all skin cancers (excluding basal cell carcinoma) 6 weeks after the first case of COVID-19 in the Netherlands. In comparison, combined diagnostic rates of all other cancer types (excluding skin) fell by a lesser 26% in the country.

A similar pattern has emerged in Australia. The Cancer Council has estimated that during the first 6 months of the pandemic in 2020; in Victoria alone, 2500 cancer diagnoses were missed [8]. This data is based off a 10% reduction in pathology notifications and the number of diagnostic procedures performed being down 30%. The state of Victoria suffered through a mammoth 282 cumulative days of ‘stay-at-home’ orders [9], raising the question of the relationship between high stringency restrictions and reduced cancer diagnosis rates. The population group in Victoria with the largest decrease in diagnosis rates was older men, living in high socioeconomic areas [8]. Again, not all populations or cancer types were affected equally. It was predicted that most of the cancers missed were head and neck, prostate, breast and melanoma [8].

Roseleur’s [10] analysis of an Australian nation-wide general practitioner database MedicineInsight and the national Medicare Benefits Schedule (MBS) service reveals interesting insights into the prevalence of screening skin checks and skin cancer diagnoses during the pandemic. There has historically been a consistent peak in skin checks and diagnoses of all types of skin cancer during the first quarter of the year (the Australian summer). In the first quarter of 2020, this peak did not occur. The number of recorded skin checks was actually below that of the fourth quarter of 2019. The skin cancer diagnosis rate in the (usual) peak period was 20% lower in 2020 for basal cell carcinomas and squamous cell carcinomas compared to that observed in previous years. Of particular concern was the greater 32% drop in melanoma diagnoses, as this type of skin cancer is highly aggressive and quick to metastasise [10].

The evidence is clear that diagnostic rates of cancers have fallen during the pandemic across all corners of the globe. The matter which remains is to unveil contributors towards this decline as the first step in mitigating these barriers for the future.

### Barriers to Diagnosis

So, why? Why are we missing so many diagnoses? The missing diagnoses could be attributable to the roadblocks at almost every step of prevention and detection of cancers since the pandemic begun, with interruption of primary, secondary and tertiary prevention programmes [7]; disruption of cancer screenings; re-deployment of staff to critical care causing delays in diagnostic scans and procedures; and the abandonment of non-emergent evaluations [11].

The switch from face-to-face to telehealth in primary care may have a role to play in the reduced diagnostic rates of some cancer types with diagnosis procedures that rely heavily on subtle cues picked up during an in-depth physical examination. For example, poor-quality photographs shared by the patient are likely insufficient to detect skin cancers, lesions in areas which are infrequently checked by the patient, such as the back, are likely to be missed, skin biopsy cannot easily be taken [12] and the patient is unlikely to be able to describe the features of their breast lump which can only be ascertained on palpation.

Individuals with non-specific cancer symptoms have felt obstructed in the process of getting a specialist consult [7]. A systemic review by Alkatout [13••] on the effects of COVID-19 on multinational cancer programmes has unveiled a high cancellation rate of diagnostic biopsy procedures of up to 71% for breast cancer, 79% for colon cancer and 58% for lung cancer [14]. This has resulted in a reduction in histopathological and cytological investigations. In Belgium, de Pelsemaeker [15] cites a reduction histopathology workflow of up to 72% in 2020, compared with the previous 3 years. The, albeit necessary, reallocation of staff and resources away from oncology during the pandemic is the core instigator of these barriers.

### Abandonment of Cancer Screening Programmes

Cancer screening programmes, which aim to detect cancer in the asymptomatic population, have been suspended, or experienced a lack of uptake across all age groups [16•]. Countries with a greater prevalence of COVID-19 have suffered the greatest decline in screening programme rates [13••]. This sparks concern that many cancers that could have been detected in the asymptomatic phase with these programmes will now be diagnosed when they are at a later, symptomatic phase, which is more aggressive and difficult to treat. Simulation modelling analysis of the Canadian Cancer Registry has predicted that the interruption of breast cancer and CRC screening programmes in the country for six months would result in a 14% drop-in diagnosis rates for these two cancer types alone. This includes missing the early diagnosis of 19,000 adenomas and CRCs in Canada [17].

In the USA, the National Breast and Cervical Cancer Early Detection Program provides uninsured women with low incomes access to cancer screening [18]. During April 2020, the total number of screening tests administered dropped by 87% for breast screening and 84% for cervical screening, compared to the April average for the five previous years [19]. The greatest declines were among American Indian/Alaskan Native women for breast screening (98%) and Asian Pacific Islanders for cervical screening (92%). It is important to note that women from racial minority groups suffer a disproportionate burden of cervical and breast cancers, with higher incidence and mortality rates than white women [19]. Women of colour are also more likely to be diagnosed with triple negative breast cancer, a more aggressive form that is associated with a poor prognosis. Therefore, the reduction of cancer screening rates is particularly worrying in minority groups.

In the USA, a cohort study by Chen [20] further differentiates the decline in cancer screening rates during the early pandemic by SES. While declines were suffered by all SES groups, the most dramatic reductions in enrolment for screening programmes were observed in individuals in the highest SES index quartile. While the threat of decreased screening is worrying across the board, it is interesting that in 2020, there was a narrowing in the disparity in cancer screening by SES. Chen [20] predicted an overall reduction of up to 90.8% for breast, 79.3% for colorectal and 63.4% for prostate cancer screening. The rates did recover in subsequent months, and it is estimated that the break has created a deficit of 9.4 million unscreened people.

Australia’s three national screening programmes for breast, bowel and cervical cancer have suffered similar blows. Each programme was affected to varying degrees; with breast screening paused for a number of months, bowel screening continuing as an at-home test and cervical screening continuing as in-clinic test. Given this, it is interesting to compare the participation rates between the programs. All programmes suffered reduced uptake [21], which may signify that it was in fact a shift in the population’s approach towards healthcare that caused the largest disruption. With such widespread media coverage and political attention directed towards COVID-19, most members of the public were likely preoccupied with the threat of the virus, monitoring themselves closely for any COVID-19 infection symptoms, while paying little attention to more subtle, early symptoms of cancer. The uncertainty of a constantly evolving situation lends itself to a ‘panic mindset’ in which one’s focus shifts from future concerns, such as screening for asymptomatic cancer, to surviving the more tangible, present threat. Disruptions in flow of everyday life may have also thrown off an individual’s vigilance in tracking the time between their regular screening intervals. With this context considered, let us compare how each screening programme fared.

Between late March and early April 2020, all BreastScreen services were paused, with only 1100 screening mammograms performed on April 2020 compared to 74,000 on April 2018 [21]. As seen in the US, minority groups in Australia were disproportionally disadvantaged by changes to screening programmes. For example, remote communities that access breast cancer screening through a mobile bus were not visited for a period in 2020 in order to reduce the risk of COVID-19 transmission [22•]. At an individual level, this caused significant psychological distress, with an affected individual stating, ‘I found a lump but all BreastScreen services have been cancelled because of COVID-19. I’m really worried it might be cancer’ [21]. Following the instalment of adaptive measures, including modified mammogram positioning techniques to allow for physical distancing [23], screening was recommenced on late April 2020. This quick adaption to the everchanging circumstances allowed 83% of the average number of screening mammograms of previous years to be met by June [21]. The number of mammograms increased each month throughout the remainder of 2020, returning to pre-COVID levels by September 2020 [21]. This recovery of the programme gives ‘a visible sense of reassurance’ [23] to affect individuals at a time of heightened global anxiety of ill health.

The Australian bowel cancer screening programme includes a mailed immunochemical faecal occult blood test kit (iFOBT). This ‘at-home’ programme with a dedicated pathology service continued throughout 2020 without disruption, allowing us to observe how screening participation rates behaved during the pandemic, with the confounding factors of needing to attend a healthcare centre or pauses to the distribution of the screening programme removed. The only remaining barrier to participation in this programme was the individual’s attentiveness. When comparing participation rates during each month of 2020 with that same month in 2019, the participation rates were lower in 2020 for all but 2 months [21]. The fluctuation in screening participation did not clearly correspond with the severity of COVID-19 restrictions during that month. The next barrier to consider is the process of further investigating via colonoscopy those who tested positive to the iFOBT. In Australia, there was pre-pandemic concern over the lengthy wait-times for iFOBT-positive individuals to receive a colonoscopy [24], with a national median of 55 days discovered during the most recent review in 2018 [25]. On top of this history, wait times for diagnostic procedures have been further prolonged during the pandemic with a substantial reduction in diagnostic procedures during the early pandemic [11]. Initial restrictions were introduced in Australia on March 2020 and by April 2020, the number of colonoscopies and sigmoidoscopies performed in the public health sector decreased 55% from the previous month [24]. While the number of investigations performed did improve in May and June, they remained 36% and 15% lower than the rates in March. While it is fortunate that the ‘at-home’ style of the bowel cancer screening programme allowed it continued uninterrupted throughout the pandemic, the programme yields little value if the individuals it identifies as being at a higher risk of bowel cancer are unable to undergo the further diagnostic procedures necessary to make a diagnosis.

While the National Cervical Screening Program (NCSP) was never officially paused, screening test rates decreased in 2020 [21]. This is most likely due to the nature of the recently upgraded screening programme from 2-yearly cytology to 5-yearly human papillomavirus (HPV) testing. As more than 2 years have elapsed since this transition, the only women expected to be screened in 2020 were those who were overdue or never screening. This relatively small number of women renders it difficult to quantify the singular role that COVID-19 played in reducing 2020 screening rates. It is likely that the transition of recommended screening test times reduced the statistical impact of the pandemic on the programme. The pandemic conditions of 2020 also made it increasingly difficult to recruit under-screened women when attendance at a clinic was required for testing [24]. Home-based self-collected tests following a telehealth consultation is an emerging practice, following the initiative from a Melbourne laboratory [26]. There is hope that flexibility demonstrated in creating a programme accessible to all women will both encourage uptake by under-screened women in Australia and provide an example across the globe of resilience in the healthcare system during this time.

In our post-pandemic recovery, we will be faced with the challenge of correcting the disruptions caused by paused screening services. The general population should be well acquainted with some of the control measures adopted to allow cancer screening programmes to resume, such as the use of personal protective equipment, COVID-19 risk assessment of participants with a screening questionnaire of recent health and travel, frequent disinfecting and physical distancing. It is reasonable that we will be able to overcome minor disruptions through catch-up programmes that prioritise those overdue for screening. However, COVID-19 has caused a far more substantive disruption: the sheer number of people who are overdue for screens is unlike anything the public health sector has seen before [24]. It is unclear what the impact of the extended screening intervals will be for these people, and it will be a challenge to overcome public reluctance to engage in cancer screening programmes. Media campaigns such as ‘Cancer screening saves lives’ by the Australian Department of Health is a leading example of the public health advocacy required at this time. While a national inquiry is being undertaken by Cancer Australia into the potential of lung cancer screen [24], the feasibility of allocating the resources towards this is questionable in light of the current pandemic-related strain on the healthcare system. Once current programmes are back on track, we can only then look towards expanding.

### Reluctance to Access Healthcare

Patients have grown reluctant to seek non-emergency medical care during the pandemic. A study by Cancer Research UK [27] has revealed that 45% of people with potential cancer symptoms did not contact their doctor during the UK’s first wave of the pandemic (from March to August 2020). Answers typically involved themes of ‘not wanting to be seen as someone who makes a fuss’ [27] or not wanting to ‘put extra strain’ [27] on the healthcare system during the pandemic. Others cited fear of contracting COVID-19 during their visit, and out of those who did visit their doctor, one third did not feel safe from COVID-19 when doing so.

Analysis by Cancer Australia [24] on the rates of utilisation of cancer services and procedures using data from the Australian MBS suggests that despite these services being classed as vital by the Department of Health which the recommendation that they remain available throughout the pandemic, utilisation dropped. Cancer Australia conclude that this is also largely due to patient reluctance. This hypothesis is supported by the recordings of anonymous calls to a cancer support service in Australia. Callers verbally reported self-election to postpone or cancel appointments due to similar fears expressed by those in the UK, e.g. ‘I’ve noticed some changes to my skin recently that I probably need to see GP about but I'm putting it off as I’m really worried about going there because of COVID’ and ‘I want to cancel my hospital appointment as don’t think it’s worth the risk at the moment with COVID-19’ [28].

Certainly, in some groups, this lack of engagement may be due to economic instability with periods of reduced work or unemployment during lockdowns. The acute exacerbation of pre-existing socioeconomic inequities is likely to make it harder for lower SES individuals to prioritise long-term health and engage with preventative health services. There is also concern that the disparity in access to health services faced by high-priority groups, such as indigenous populations in Australia, could be exacerbated by the pandemic.

The COVID-19 pandemic has “important implications for the pursuit of quality oncology care and achieving equity and justice in cancer care settings. These social and economic implications are potentially more enduring and impactful than the immediate biophysical consequences of the COVID-19 pandemic [2•]

- Dr Alex Broom, 2020.

### Future Burden

**Fig. 1:**
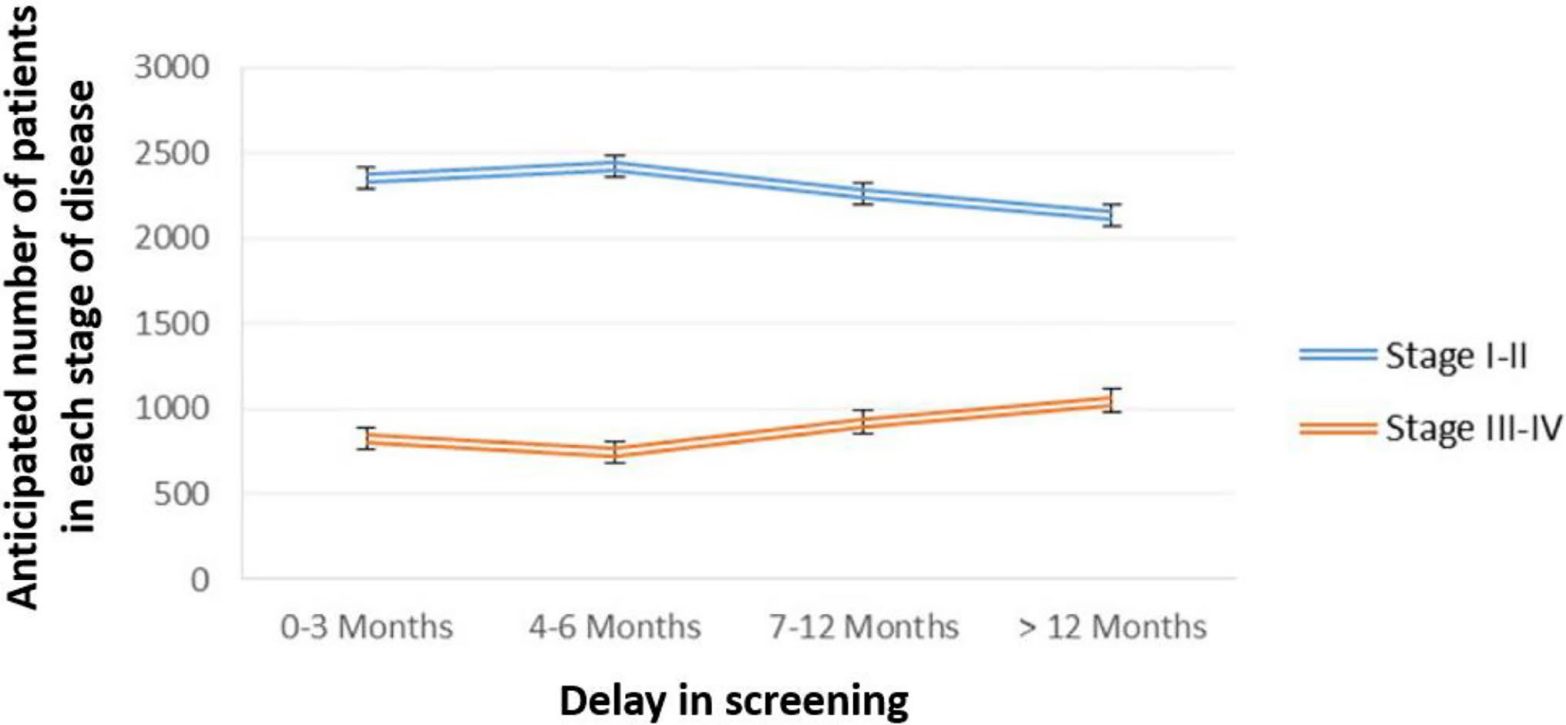
Estimated colorectal cancer progression due to delayed diagnosis, at various intervals. Reproduced, with credit to Alkatout et al. [13••] for creation, based on data from Riccardiello et al. [28]

The present reduction in cancer diagnostic rates leaves a large undiagnosed population. Once a sense of ‘normality’ returns with the general population once again comfortable to access primary healthcare and the complete recommencement of screening programmes, there is likely to be an influx of new cancer diagnoses. The scary thing about this impending tsunami of diagnoses is that most detected cancer will be in later stages, making the disease less likely to be curable, inferring worse prognoses. Patients with advanced-stage cancer will also be more likely to experience debilitating symptoms of their disease. Ricciardiello [28] concluded that screening delays greater than 12 months will allow for undetected CRC to progress, causing a 7% increase in the detection of CRC tumours at an advanced stage (Fig. 1).

A shift in disease-stage at initiation of treatment will carry a significant economic burden at a population level. Degeling [29••] has developed an inverse stage-shift model which extrapolates the impact on expected deaths, life years lost and healthcare costs of changes in the distribution of cancer stage due to pandemic-related delays in initiating treatment. This model is based on weighing stage-specific outcomes and has been developed into a publically available online calculator [29••] into which data for any country or cancer type can be entered (REF). Analysing Australia’s four most common cancer types (breast, CRC, lung and melanoma), Degeling [29••] predicts that a 6-month delay in diagnosis and treatment would ensure excess mortality and healthcare costs of 349 deaths and US $46 million over 5 years in Australia.

A deficit in screening-detected cancers is estimated to persist for at least a year after screening programmes resume [17], as it is predicted that even after services are running at full capacity again, it will take at least another 1 to 2 years to clear the backlog of missed cancer screenings [16•, 30]. When screening resumes, new cancer diagnoses will come as a sudden spike, amounting to huge pressure on the healthcare system and the professionals working within it. The more we delay these services now, the more hurt there will be in the future.

**Fig. 2:**
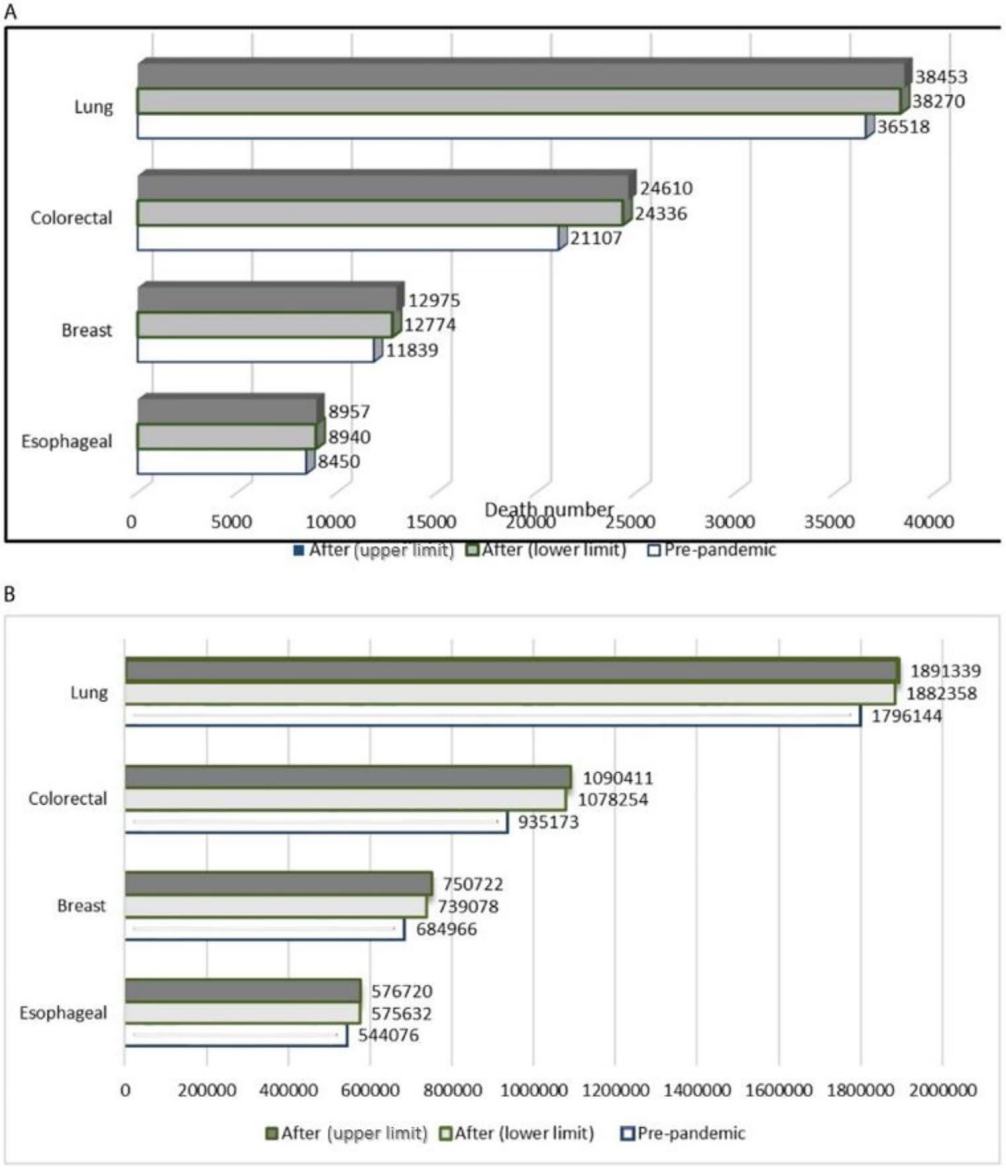
Estimated cumulative number of deaths due to breast, colorectal, lung, oesophageal cancer up to 5 years after diagnosis, **a** in the UK and **b** global total, reproduced, with credit to Alkatout et al. [13••] for creation, based on data from Maringe et al. [16•]

Indeed, the number of additional deaths caused by the interruption of cancer care services is expected to rise within the next 5 years and continue well beyond 2029 [17]. Cancer mortality rates are predicted to increase by 15% for CRC and 9% for breast cancer over the next 5 years in the UK [4]. A national population-based modelling study by Maringe [16•] used data from the English National Health Service (NHS) cancer registration and hospital administrative datasets to predict the impact of diagnostic delays on survival. Across the four cancer types of breast, CRC, oesophageal and lung cancer, Maringe foresee 3291–3621 additional deaths from cancer within 5 years from diagnosis (Fig. 2a). This corresponds to as many as 63,229 years of life lost (YLL) in the UK alone. Maringe’s predictions have been extrapolated by Alkatout [13••] to a global scale (Fig. 2b), using data from the International Agency for Research on Cancers [31] on the total number of cancer deaths in the UK and worldwide.

## Disruption to Oncological Treatment

Reprioritising the limited available resources in the face of a pandemic has caused delays in the provision of standard care, with a significant reduction in active cancer treatment rates. Globally, the World Health Organisation (WHO) have found that 122 countries, out of the 163 countries investigated, have had disruptions in the delivery of non-communicable disease services. Cancer makes up a big proportion of such diseases and in Europe, where the burden of COVID-19 has been felt particularly significantly, and 1 in 3 countries had partially or completely interrupted cancer care services early in the pandemic [4]. In the UK, around 40,000 fewer patients than normal started cancer treatment in 2020. In comparison to Europe, Australia has suffered a smaller burden of COVID-19 cases yet has still been subject to travel restrictions, shortages in drug supply from international suppliers and a strained health system. As such, between January and June 2020, there was a 18% decline in cancer treatment [8].

Those patients for which surgery was planned have been most significantly affected. Some have had their treatment plan altered to chemotherapy or radiation therapy, or their surgery simply delayed. Globally, nearly 38% of cancer surgeries were cancelled during the first 12-week peak of the pandemic from late January to early April 2020 [32]. Nepogodiev [32] estimates that theatres would have to increase their normal surgical volume by 20% over a median 45 weeks after the pandemic to clear this backlog. Yet, this data only looks at the first 12 weeks of disruptions and there has been variable yet ongoing disruption to surgeries for almost 3 years now. The implication of these delays is clear with Johnson’s [33] systemic review and meta-analysis of surgery delays concluding that a 12-week delay in surgery is associated with decreased overall survival in breast cancer (particularly stages I and II), lung cancer and CRC. The UK’s Royal College of Surgeons agrees that it could take several years working overtime to clear the backlog [34]. There are also fears that there will be a wave of compensation claims from cancer patients whose disease has progressed while waiting for surgery [35].

**Fig. 3:**
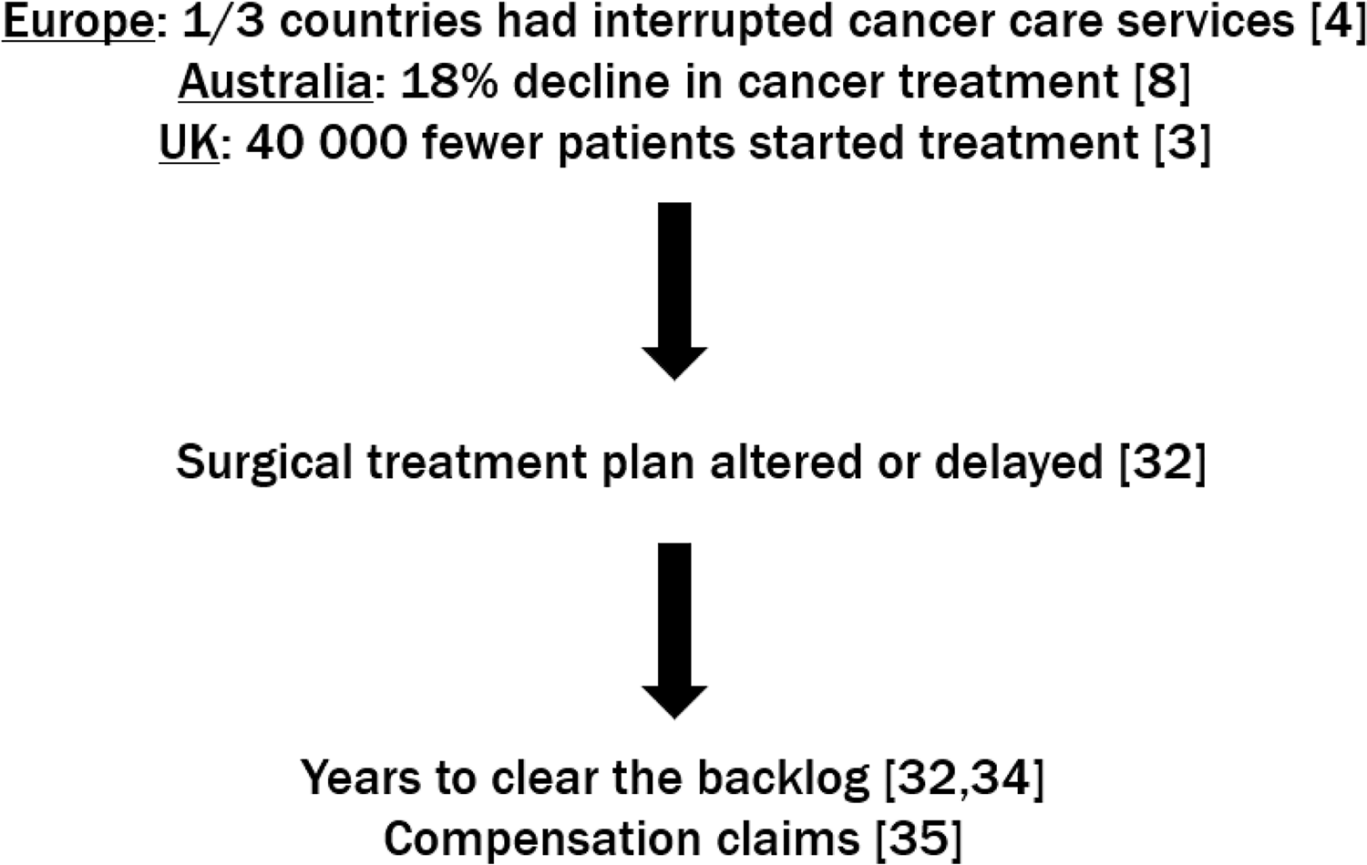
Roadmap demonstrating the flow-on effects of interruptions to oncology services during the COVID-19 pandemic

Despite patients being offered systemic anti-cancer treatments (SACT) in the UK, there has been a reduction in active treatment. On analysis of the records of eight major hospitals in the UK during early 2020, Lai [36] discovered a 60% reduction in SACT appointments from pre-pandemic levels (Fig. 3).

Oncologists have had to display profound adaptability and critical reasoning in developing an individual patient’s cancer treatment plan during the COVID-19 pandemic. It has been a display of the art of finely balancing the benefits of a treatment to the patient’s disease course with the potential risk of COVID-19 infection. There is evidence that the pressures of the current climate have forced clinicians to more rapidly identify and withdraw the provision of non-essential drugs, such as adjuvant and preventative treatments, from patients’ regimens [37, 38]. Other adaptations include reducing the frequency of immunotherapy infusions or switching a patient to oral SACT rather than intravenous treatment to reduce visits to hospitals and treatment centres. Broom [2•] sees this as an experiment that may help the oncology community reduce the use of superfluous care and recognise ‘unnecessary and even damaging practices’ of the past – a hard reset of the norms of clinical practice.

## Adaption of Medical Practice

### Uptake of Virtual Consultations

The pandemic has ‘forced a fundamental rethink of health care delivery’ [39], prompting huge shifts in medical practice. The uptake of telecommunication has been widespread and will likely continue to proliferate through healthcare systems. However, there is limited evidence underpinning the delivery of remote care and how the quality of communication and support compares with face-to-face delivery. There are accounts of patients expressing these same concerns as to whether virtual delivery of healthcare will compromise the quality of the care they receive [40•].

From a monitoring perspective, a lack of face-to-face engagement in an atmosphere of global-heighted uncertainty has created increased anxiety about missed recurrence in some cancer patients. Aside from ensuring quality of care during the virtual shift, the issue of access to healthcare equity again rears its head. Patients’ socioeconomic factors (including class, education level, language, age and race) all feed into the comfort and ease with which the patient will access this technology [2•]. Virtual consultants also rely upon stable internet connection and updated electronic devices that are available to some but are not equally distributed across the population. It is postulated that at the tipping point of virtual consults being core to oncological practice, we will see further regression of marginalised groups [2•, 41].

Telecommunication does, however, provide a way for patients to remain engaged in their healthcare during periods of ‘stay at home’ orders. Chen [20] demonstrated the benefits of this tool in their cohort study, which showed through multivariable analysis that the use of telehealth was associated with higher cancer screening rates than the general population during the pandemic. While the current standard of telecommunication may not meet all needs, this virtual shift has kickstarted more creative ways of thinking about service delivery which can be utilised to support future patients who cannot travel due to extensive distances, being too unwell, or having no supports available to assist them.

It is certainly harder to form a strong doctor-patient relationship over video interview. Dr Stephanie Archer, a health psychologist heavily involved in psycho-oncology research, coins this vital relationship the ‘therapeutic alliance’ [42]. Telehealth no doubt impacts on ‘moderation, trust, decision-making, and adherence’, which will be felt particularly significantly in the field of oncology, as patient visits are frequent, and the therapeutic alliance is imperative for determining goals of care. Beyond this, an oncological consultation serves a ‘social, moral, and ritual significance’ as mentioned by Broom [2•]. These dimensions of the interview do not seamlessly translate in a virtual consult.

The effective removal of face-face contact from every interaction will have unmapped and unknown effects—affect moderation, trust, decision-making, adherence—may make things better or worse—but I am worried it will be accepted as equivalent for expediency” [42]

- Dr Stephanie Archer, 2020.

As such, a large majority of communication is non-verbal and this nuance is easily missed over video call. Visual cues are essential in the formulation of a judgement as to how well the patient is. Without this interaction, the oncologist is left reliant on the patient’s self-report of how they are coping with their burden of disease and treatment. The subtleties the oncologist picks up in observation of family members discussing amongst each other often reveal the patient’s true wishes and goals of care, but this is also less detectable with telecommunication.

At particular stages of a patient’s cancer journey, the practitioner and the patient have different priorities for the consultation. Thus, different communication modalities may yield the most value at different stages of care. Koczwara [41] suggests that telehealth may be most optimal for long-term cancer survivors, as the purpose of the consultation is less on active treatment, and more on surveillance and aiding the transition to normal life. Greenhalgh [43] pre-pandemic research into the use of video consultations supports the understanding that this medium is better utilised when the clinician and patient have already developed a strong therapeutic relationship.

Communication and meetings between healthcare professionals in multidisciplinary teams have largely shifted to telecommunication. While this was a necessary adaption to social distancing requirements, it has come with many unforeseen benefits. The ability for clinicians to work remotely to attend both virtual meetings and conferences allows for time-management benefits and increased productivity, with a reduction in travel time between healthcare facilities [11]. International conferences being delivered online opens the floodgates to the international sharing of knowledge and experience, with more professionals able to attend virtually than exclusively those who would have been able to travel. This can only benefit our field and patient care in an increasingly globalised era.

All of the possible uses of digital communication in the healthcare sector are yet to be realised. It would be easy to have a shared consultation with both the patient, general practitioner and oncologist present, speeding up the transmission of information between doctors and reducing the risk of important information getting lost in communication. It would also be possible to have members of allied healthcare team and family members from distant locations simultaneously present at such consultations.

Yet, we must not simply accept primarily virtual communication as the new standard of care going forward, without first scrutinising its effectiveness and building a stronger evidence base to support or negate its use. While many believe the shift is inevitable, let us ensure that it is a smooth one by providing additional administrative support and training opportunities to facilitate it.

## The Patient’s Perspective

### Mental Health Vulnerabilities

The COVID-19 pandemic poses multifaceted psychosocial challenges to all those affected by cancer, and a growing mountain of evidence has shown how the pandemic continues to affect the mental health of all human beings [44]. To contextualise this, during 2020 in Australia, there was a dramatic increase in calls from the general population to mental health services, e.g. calls to mental well-being support service, Beyond Blue, rose by 42% during 2020 [45]. When COVID-19 restrictions were first introduced in Australia on March 2020, there was a spike in mental health-related PBS prescriptions. General practitioners also reported a 30% increase in mental health consultations throughout 2020. The demand for mental health service only continued to grow in 2021, with the support service Lifeline registering multiple historical record high daily call volumes in the August–September period alone. The massive volume of calls were up 14.1% and 33.1% from the same period in 2020 and 2019 respectively [45]. The oncological community should be aware of a mental health crisis within the general public as it is likely that many of their future patients will require more intensive supports to meet their psychosocial needs.

Cancer patients may be more vulnerable to the mental health impacts of the pandemic due to the pre-existing psychological burden of a cancer diagnosis or survivorship. The common, universal thread woven throughout oncology patients’ reflections on care during the pandemic is a feeling of loneliness [42, 45, 46]. For many, both international and interstate border restrictions prohibit cancer patients’ ability to see friends, family and carers, even at the end of life. This social isolation and uncertainty at a time of psychological distress certainly compounds the burden of disease.

Edge et al. [40] explored the significant and enduring psychosocial impacts of the pandemic on cancer patients, survivors, and carers in New South Wales (NSW), Australia. Data from Cancer Council NSW supportive services was assessed for service demand, caller distress levels and content themes, using inductive conventional content analysis of call notes and deductive directed content analysis on online forum posts. Psycho-oncology support service demand peaked on March 2020, which correlates with the beginning of virus control measures put in place by the Australian Government [47]. Self-reported distress of respondents peaked on May 2020, with an average distress level of 8/10.

Edge’s qualitative analysis found that 53% of callers expressed psychological distress due to feelings of isolation, with one caller stating, ‘I feel totally alone. I could cope with the cancer or COVID-19, but not both together. It’s too much’. The burden of this isolation is shared with carers who have been faced with limitations on healthcare visits and restrictions to travel and physical interaction with loved ones. One carer stated, ‘My partner is dying in hospital and I’m so distressed that I might not be there when he passes away. COVID has meant that I can only visit him at certain times’ [40].

The disruption of cancer services — including delayed appointments or treatments, self-elected cancellations and transition to telehealth – has invoked anxiety surrounding disease progression in patients. Twenty-six percent of patients who posted on the online cancer forum in Edge et al. [40] study expressed these concerns, with one anonymous contributor stating, ‘I was diagnosed with cancer a few weeks ago but got told yesterday that due to COVID risk my surgery has been delayed. I feel really isolated and worried about the delays and what this means for the cancer’.

It is important to note that compared to many other countries, Australia has suffered a comparatively small burden of disease from COVID-19 [48]. While the ‘uncertainty, disruption and social isolation’ has been a universal experience during the pandemic, it is likely that in countries where COVID-19 has been destructive, the psychosocial impacts on those living with cancer may have been even more debilitating. Just as we will continue to see impacts of the pandemic is many other areas, it is safe to assume that the psychosocial consequences of this trying time will endure long after the crisis.

### Perceived COVID-19 Susceptibility

Those callers expressing loneliness commonly shared a fear of perceived COVID-19 susceptibility, likely in response to public health guidelines advising patients with cancer and their carers to take extra precautions in reducing physical contact [49]. Based on evidence from previous infection outbreaks, including influenza [50], severe acute respiratory syndrome coronavirus (SARS) and Middle East respiratory syndrome coronavirus (MERS) [51], it was widely assumed by the healthcare community at the start of the pandemic that the immunomodulatory effects of both cancer and SACT would place this patient group at higher risk of COVID-19 infection and adverse outcomes. Only through enduring the course of the COVID-19 pandemic could data be gathered by cancer registries to prove or dispute this hypothesis, leaving those living with cancer in the dark for some time about their personal risk. Even as evidence began to mount in the later stages of the pandemic, many callers felt there remained a lack of clear guidance on the precautions that oncology patients and their carers should take and this group’s specific risk profile of contracting COVID-19. This has only added to the anxiety surrounding the pandemic. Indeed, information seeking was a major theme identified in 44% of posts to the online cancer forum during Edge’s [40] collection period. The need for clear, concise advice was also identified by Cancer Australia’s [22•] framework for managing cancer patients during the pandemic, which prioritises the need for improved communication of information to both patients and healthcare professionals.

The USA’s National Centre for Health Statistics have released weekly data on COVID-19-related deaths in the country from the National Vital Statistics Systems. This data is taken directly from death certificates at the state and local level and has been categorised by age, gender, race, geographic location and comorbidities [52]. Interestingly, cancer was not amongst the most frequent comorbidities of COVID-19 deaths, which included influenza and pneumonia, hypertension, diabetes, dementia, and sepsis. Malignant neoplasms were a comorbidity in 33,216 deaths across all age groups, as of 3 October 2021. This is out of a total of 700,952 deaths attributed to COVID-19, meaning that cancer was a comorbidity in approximately 4.74% of deaths. As a comparison point, the latest estimated population prevalence of cancer in the USA is 16,353,421 people [53], which makes up 4.96% of the total population of 329,470,846 at the end of 2018 [54]. A crude glance at this data would imply that the cancer population is proportionally represented compared to the general population in COVID-19 deaths. This is in opposition to the heightened fear of death by COVID-19 felt by many people living with cancer. Of course, this data does not suggest that cancer does not place an individual at greater risk of death from COVID-19, as many of these more frequently reported comorbidities may simply have a greater prevalence in the general population. The lower frequency of comorbid cancer may in part be attributable to greater precautions being taken by this group to prevent initial infection.

While oncology patients may not be at increased risk of infection with COVID-19 compared to the general population, a review by Lee and Purshouse [55] has collated evidence from national and multinational cancer registries on the risk factors for adverse outcomes following COVID-19 infection in patients with solid cancers. In summary, this review concluded that patients with cancer have a higher probability of death from a COVID-19 infection compared to those without cancer. Mortality rates from COVID-19 varied between cancer registries, from 13 [56] to 40.5%, compared to 28.5% in those without cancer [57]. The most significant risk factors for death in this group was older age, male sex, smoking history, two or more comorbidities (particularly cardiopulmonary comorbidities) and poor performance status with an Eastern Cooperative Oncology Group (ECOG) score of > 2. The oncological features most highly correlated with risk of death from COVID-19 infection were having a lung or thoracic cancer and disease being in an active or progressive phase. The majority of studies did not find a significant contribution of SACT to adverse outcomes from infection. However, there may be a correlation between recent receipt of chemotherapy and chemotherapy-immunotherapy within two weeks, and a higher risk of mortality [58]. Larger studies will be required to confirm this preliminary finding. Identification of specific risk factors will facilitate patient-specific advice regarding degree of self-isolation advisable during outbreaks, and how to mitigate the psychological adverse effects of fear and loneliness which result from blanket stay-at-home advice to all cancer patients.

### How Oncologists Can Adapt to Meet Their Patients’ Needs

The therapeutic relationship which normally exists between oncologists and their patients is universally blunted via virtual consultations. As particular cultural groups may be more inclined to process and express bad news differently, the severity of the impact of this virtual shift will differ in various regions of the world, and between individual patients in a multicultural centre. The physician should be aware of the discrepancies between how different patients are coping with the loss of face-to-face consultations, as well as a diverse range of other practical issues which their patients may be facing at this time, including employment changes, loss of income and financial hardship. Oncology patients’ perceived vulnerability to COVID-19 infection may lead them to minimise their exposure risk by discontinuing work during the pandemic, especially if they are essential workers. Fear of contracting the virus can make everyday feats, such as shopping for essential items and catching public transport to medical appointments, particularly challenging. Thirty-three percent of callers to the support service cited distress due to similar practical issues, especially at shortages in medication or personal protective equipment supplies [40].

The challenges that each individual patient is facing during this time is unique and it is imperative that their doctors ‘mobilise (their) best skills as oncologists to provide individualised and compassionate care’ [46]. Oncologists, along with the support of their multidisciplinary team, should aim to identify patients at the highest risk of disadvantage due to COVID-19 restrictions. There is potential for the pandemic to worsen pre-existing socio-economic determinants of health. This is illustrated by one caller who stated, ‘…I have to travel 40 min away for a biopsy. I have no reliable transportation at this time, and with the coronavirus, it won’t be scheduled for quite some time. I live in a very rural area… on a fixed income and can't afford to pay someone to take me. I also have no family. I called social services too but had no luck…’ [40].

It is clear that patients with cancer require additional psycho-oncological support during the COVID-19 pandemic. However, this is at odds with the reduction in availability or access to these services. The British Psychosocial Oncology Society completed a cross-sectional qualitative survey of UK professionals in 2020 [42], which revealed that pre-existing psycho-oncology support services were largely not prioritised by referring practitioners during the pandemic. Even when these services remained open, there were a reduction in referrals to them. This data is at odds with the increased demand on direct-to-consumer psycho-oncology services observed in Australia, revealing that the reduction in referrals was more likely to be based on practitioner factors than patient need. Respondents to the British Psychosocial Oncology Society survey predicted that this prioritisation of resources away from psycho-oncology at present in the UK will cause an ‘avalanche of demand’ when things ‘return to normal’ [42].

Therefore, it is imperative that we work to develop and deliver accessible support through targeted interventions that specifically meet the evolving psychosocial needs of those affected by cancer in the greater context of the COVID-19 pandemic and post-pandemic period. On a public health level, this would include the distribution of timely and reliable information by national cancer bodies, which is comprehensible to all health literacy levels. Additionally, there is a need for international education to the public to encourage seeing a primary health care provider regarding symptoms of concern and taking part in regular cancer screening appointments where they remain available.

At an individual clinician level, the oncologist should be called to action to continue providing tailored and patient oriented care, with an understanding of how a patient’s psychosocial well-being impacts their quality of life and clinical outcomes. A shared decision-making approach should be utilised in the discussions surrounding unanticipated changes to cancer care plans and the rationale behind these, in order to reduce the feeling of abandonment and uncertainty that many patients have expressed [40]. This pandemic has shown us the need to develop innovative and holistic care model that can continue throughout crisis events. We must not devalue the importance of carer support to cancer patients, within the limits of community safety. This can be addressed through providing better access to telehealth-based supportive care and family visitations on compassionate grounds.

COVID‐19 is marked by relentless loneliness and fear. These emotions are experienced by individuals, families, and communities. In the face of this, we mobilized our best skills as oncologists to provide individualized and compassionate care, while supporting each other as colleagues and friends. [46]

- Dr Reynolds, 2020.

## Medical Research

The global pandemic has had significant impacts on the conduct of medical research and clinical trials in oncology. As infection control measures were implemented, oncology trials were largely halted. The world’s focus shifted away from cancer research, taking with it financial cutbacks by many academic institutions [59], a global reduction in patient enrolment in oncology trials [60•] and stunting the activation of new trials [61]. These changes are particularly detrimental for patients with rare cancers or patients who have progressed on all available the treatment lines, who invest their hope in new experimental therapies where there is a lack of standard treatment options [2•]. The desperate need for research to continue has forced “the largest change in clinical trial conduct since the start of modern oncology clinical testing” [62], with the introduction of patient-centred, decentralised study models.

At the advent of the pandemic, regulatory bodies across the globe stepped in to enforce restrictions on the conduct of medical research. In the USA, guidance from Institutional Review Boards (IRB), the Food and Drug Administration (FDA) and cancer professionals’ organisations, suggested limiting operations to ‘critical research’ [63]. What this term meant was left up to the interpretation of the organisation running the research [11]. The decision to continue a clinical trial is made after assessing the study’s benefits, and the potential COVID-19 exposure risk for vulnerable patients and staff. For this reason, trials that investigate aspects of care other than survival benefit were largely halted. Interventional studies have also been stopped if the investigator agent increases immunosuppression or pulmonary toxicity risk [60]. Surveillance from the Cancer Research Institute reveals that the total number of suspended oncology trials peaked on May 2020 [64]. In the UK, this amounted to the suspension of 95% of previously running cancer trials, according to Cancer Research UK [59]. Non-interventional trials or interventional trials that did not involve active treatment but had a supportive care, diagnostic or screening purpose were not seen as ‘critical’ and much more likely to be suspended [11]. Just as psycho-oncology was abandoned, we again see the abandonment of supportive care during the pandemic, with the gaze narrowed to active treatment alone. While this is a safe and logical adaption in the current circumstances, this move can only add to the future burden of unmet psychological need and missed diagnoses. However, trials involving paediatric oncology patients were affected to a lesser extent, given evidence that this population group was less likely than adults to develop severe COVID-19 infection [65].

Unsurprisingly, there has been a dramatic decrease in the number of new cancer clinical trials activated. Data from an international commercial clinical trial platform (Medidata Enterprise Data Store) operating across 91 countries reveals a 60% reduction in the number of launches of trials studying cancer therapies between January and May 2020, compared with pre-pandemic levels [61]. Some of this reduction may be accounted for by the prioritisation of reactivating pre-existing studies, over activation of new ones [59]. Another contributor is reduced availability of in-demand health care professionals. Many members of this group have been redeployed to take on clinical roles in frontline healthcare services which have been under substantial pressure during the pandemic [66]. Therefore, reprioritising clinical work over the planning of new clinical trials has further stumped research progression [67]. Interruptions to the supply chain of investigational products during the pandemic have also been shown to be a contributor towards delays in medical research [42, 68]. Retarding the development of new cancer therapies and ‘the momentum of scientific progress’ [61] exemplifies the long-term, indirect health effects which the pandemic may have on population morbidity and mortality.

There has been a global reduction in patient enrolment in clinical trials [60•], based upon records from internal databases of large research institutions and statements of from key stakeholders [69]. The American Society of Clinical Oncology (ASCO) accounts a 50% decline in enrolment during the early pandemic [69], with Acorn Al Labs also reporting a dip in patient enrolment in both ongoing and new trials [59]. This pattern reflects broader population trends towards a resistance to interact with health care systems [24, 27, 28]. Enrolment is further made difficult by cuts in the volume of outpatient clinics to adhere to physical distancing and density requirements. Reductions of up to 50% of patients seen in typical week, reported by a US academic medical centre, smother the recruitment opportunity to inform and enrol new patients in clinical trials [11].

To continue medical research safely in the face of the pandemic, we have seen adaption to a new framework of protocols and procedures, demonstrating that ‘even in a time of despair… innovation can emerge’ [11]. The net shift has been away from a trial site-centred process, to a patient-centred approach [62, 69]. The uptake of ‘fully decentralised virtual approaches’ to clinical trials [69], as described by the European Society of Medical Oncology (ESMO), has allowed for the majority of previously-halted studies to be recommenced [64]. This model permits oncology trials to continue during pandemic conditions through allowing clinical coordinators and research staff to work remotely during periods of stay-at-home orders. Decentralised studies have also been shown to improve workflow efficiency, patient experience and reduce trial running costs [66].

In the USA, these adaptions have been steered by the FDA’s guidelines on the conduct of clinical trials during the pandemic [63]. The following changes have paved a model which could become standard practice or be swiftly reintroduced during future pandemic conditions: (1) Procedures which can be shifted remotely, such as consenting, have been, e.g. it is now commonly practiced to email out blank consent forms, hold the consenting discussion over the telephone and have the forms signed electronically signed [60•]; (2) the trial drug can be delivered directly to the patient at home [62]; (3) during the monitoring phase, site visits can be reduced by using electronic medical records for follow-up questionnaires and self-reported data [69], as well as the use of wearable devices which provide the researcher with a live feed of information from the patient at home [66]. (4) Many assessments can be carried out closer to home, such as allowing patients to have imaging and laboratory samples taken at local facilities rather than the trial centre [62] and having physical assessment performed by the patient’s primary care physician [71].

In Australia, the Therapeutic Goods Administration (TGA) has made similar suggestions in their guidance on clinical trials statement, suggesting researchers consider alternative models such as decentralised ‘tele-trials’ with remote data collection to minimise exposure risks [47]. The UK’s Medicines and Healthcare products regulatory agency (MHRA) [72] similarly released their ‘Managing clinical trials during coronavirus’ guidelines which support the use of remote monitoring. However, MHRA also raise concern around potential confidentiality issues when patient’s electronic records are accessed off site [72]. This concern is shared by professionals across the globe, with both Italy and Switzerland having strict data protection rules which prevent the use of electronic health record and uptake of remote monitoring [69]. Once again, all of these guidelines act only as non-binding recommendations, with much of the decisions left up to the discretion of the researchers.

Some studies may lend themselves better to a hybrid between the traditional and patient-centred approach. If in-person monitoring is absolutely necessary, a flexible schedule will be required to allow for periods of intensified travel restrictions whereby participants may not be able to travel to the clinic. Of course, there will need to be deep consideration as to whether this ‘hands-off’ approach is safe for patients, by weighing up the potential risk of the individual investigated agent. At times, physicians may still need to conduct patient visits to adequately evaluate treatment tolerance, safety, adverse effects and to collect samples for laboratory studies. The consideration here should be as to whether this visit increases the patients’ risk of contracting COVID-19 above the standard-of-care.

When redesigning a study to proceed within pandemic conditions, consideration must be given to which aspects of clinical trials are essential for efficacy and safety assessment, and which common practices are actually low yield [62]. For example, the routine screening of basic patient parameter such as height and weight were abandoned by many trials during the pandemic to reduce site visits, with no obvious detrimental impacts on the safety of the trial [66]. Furthermore, many trials forewent screening investigations such as echocardiograms in patients with no history of or risk factors for cardiovascular disease [67], as the benefit of completing this investigation was so little compared to the risk of COVID-19 exposure.

At an organisational level, the logistics of planning and conducting medical research have simplifier over the course of the pandemic. For the first time, FDA and IRB submissions of proposed clinical trials also became electronic. This change is likely to stay due to marked increases in workflow efficacy [11]. One might argue that this change lags behind the enthusiastic embrace of technology we have seen in other spheres of life over the past decades, but it is likely that the transition to electronic submissions would not have occurred for many more years to come without the pressure to adapt posed by the pandemic. Given that the original protocol of studies which commenced pre-pandemic had to be violated in order to allow the study to continue during the pandemic, deviation reporting processes have largely been relaxed [66]. Cancer Research UK suggests reporting only major protocol deviations in individual deviation reports and grouping all minor deviations together into a weekly report [73]. This reduces the burden of administrative work in the face of countless protocol violations and helps streamline trial efficiency.

The next question is, what is the impact of these operational changes on the quality of research produced? Positively, the uptake of remote monitoring in clinical trials leads us to consider new research inquiries, such as exploring the effectiveness of using remote patient monitoring in oncology. This is something that is unlikely to have been considered if the pandemic had not pushed us in this direction. ESMO postulates that the accelerated adoption of new operational approaches in light of the COVID-19 pandemic as well as ‘tighter collaboration among all clinical trial stakeholders’ may allow patients faster access to emerging treatments in the near future [69]. Marcum [11] agrees, reporting significant increases in the efficiency of workflow under the new US guidelines due to relaxed reporting requirements. Streamlining clinical trials through the utilisation of technology, reduction in site visits and minimisation of bureaucratic barriers not only simplifies and fast tracks the research process but minuses the burden placed on all involved. The grounds which have been made in improving study design should not be discarded off after the pandemic is over.

Moving into a post-pandemic world, a proportion of studies would be ideal candidates for the continued use of patient-centred trials. For example, the choice of a fully decentralised trial may be most appropriate for non-interventional studies, such as those tracking long-term follow-up [69]. Interventional studies may call for a hybrid approach, giving consideration to the phase of the study, route of intervention, safety profile and study endpoints. This approach will be particularly appealing in the immediate post-COVID era as many people continue to work offsite.

The practical demands placed on clinical trial patients has evolved throughout the pandemic. Continued use of the new, decentralised approach to trials will reduce the burden placed on the patient in terms of time, travel, costs and stress [69]. The approach also allows the trial to be accessible to a broader population by limiting practical issues of geographic distance and transportation difficulties. There is hope that this could help tackle the lull in patient enrolments. Furthermore, Sessa et al. [69] suggests that broadening eligibility criteria to accurately mirror the population likely to use the intervention will assist in boosting patient enrolments. Restrictive exclusion criteria which are not based on sound scientific justification should also be abandoned.

The COVID-19 pandemic has forced a dramatic shake-up of the medical research sector, the rapidness of which has never been seen before. We have seen adaptions at all levels of the system; from trial design, to conduct, reporting, patient enrolment and experience. While these changes come out of a desperate need to persist with research within the challenging circumstances, many have shown to be more efficient than the pre-pandemic norm and should be retained as we move into a post-pandemic future.

## Future of Oncology

### Recommencement of Services

In the immediate aftermath of the pandemic, the priorities within the field of oncology will be to recommence screening, diagnostic, and treatment services. As we move towards unveiling the ‘new normal’, policy-maker decisions must be led by a drive to ensure equity and social justice in the provision of oncological care. It is predicted that resources will continue to be rationed throughout future waves of COVID-19, reaffirming the need to standardise what is considered critical and non-essential care in the oncological field [2•].

If the COVID-19 pandemic has taught us one thing, it is that early detection of disease is key. This principle still applies to non-communicable diseases such as cancer. Therefore, efforts to address the cancer screening deficit associated with the pandemic are required at a public health and health service level, in both the public and private sector [13••]. These policy interventions will be particularly important in managing the backlog within routine diagnostic services [16•]. When services are back to running at full capacity, it is predicted that it will take 12–24 weeks to complete the missed cancer screenings [16•, 30]. Kregting et al. [74•] modelled the effects of four different screening restart strategies on population cancer burden, mortality and capacity requirements. The best balance between these factors was found to be in the scenario with delays in screening but with all screening rounds still offered to the population.

Screening programmes and diagnostic services must adapt to using telehealth in order to allow them to continue throughout future regional outbreaks. As we are likely to see the effects of the pandemic on the healthcare system endure for some time, efficient prioritisation of healthcare resources is necessary to mitigate the enduring negative effects. There have been suggestions to take a risk-based approach to screening programme [22•]. In this model, prioritisation would be given to those at the greatest risk of an interval cancer if they were to have an extended period between screens. Another suggestion is the introduction of lower resource-intensity screening programmes. Efficient recommencement can also be achieved through primary care providers triaging patients who have missed resource-intensive cancer screening tests, while investigations with a mild resource-burden, such as the iFOBT, can be recommended for all [5]. To begin clearing the backlog of patients waiting for surgical procedures, preoperative evaluation of predictive markers may be useful in triaging the extensive list of patients [5]. An example of this would be the iFOBT for patients with symptoms of bowel cancer, prior to prioritisation for colonoscopy, as suggested by Cancer Australia [22•]. At-home screening tests in this style should be retained and encouraged throughout all future lockdowns. National screening questionnaires may be beneficial to stratify individuals’ cancer risk; however, this approach is likely to further disadvantage those with a lower health literacy. Chen [20] suggested the possible use of screening modalities that do not require a procedure. For example, if restrictions limit colonoscopies from being performed, or there is a significant wait list for this procedure, CT colonography or double-contrast barium enema could be considered, to aid in triaging patients’ need for theatre [5]. Given the known health and health economic benefits of screening programmes, we must use this opportunity to reflect on the issues of current programmes and optimise these for the future.

Making these changes at COVID-free hospitals may alleviate patients’ anxiety about contracting COVID-19 during hospital visits. This would also mean other institutions would become dedicated hospitals for patients with suspected or confirmed COVID-19 [13••]. Public health educational interventions will be necessary to inform the public about the importance of seeking medical care for potential cancer symptoms and the safest way to reengage with these services.

An increase in healthcare funds, proportionate to the severity of this situation, will be required to allow for adequate staffing and space to facilitate a speedy catchup process. Even if only patients with the most need for a diagnostic procedure or intervention are targeted, and moderate to low risk patients excluded from the first phase of the catchup process, current resources are unlikely to be sufficient to meet the needs of this group in a timely manner [13••]. Governments should seriously consider providing extra funding in order to avoid the excess healthcare costs of delaying cancer diagnosis and treatment [29].

### New Technologies and Treatments

The future of oncological treatments will undoubtedly be shaped by the rapid technological advances we have seen during the pandemic. There is hope that efficient design and manufacturing processes used in the development of COVID-19 vaccines could be transferrable to other diseases, including cancer therapies. For instance, the developers of the Oxford AstraZeneca vaccine, in collaboration with the Ludwig Institute of Cancer Research, have founded a biotechnology company called Vaccitech Oncology Limited. Clinical trials for a novel immunotherapeutic vaccine targeting non-small cell lung cancer have begun [75]. There is further research taking place into recombinant virus vaccines to be used in the treatment of late-stage prostate cancer and therapeutic vaccines to treat chronic viral infections including hepatitis B, which would reduce the incidence of hepatocellular carcinoma. The Lancet Oncology [3••] has expressed hopes that the swift approval of vaccines may pave the way for accelerating the approval process of other new drugs, including cancer treatments — of course, for this acceleration to occur, there would need to be equal amounts of international pressure and financial support.

To fill the gap left by reallocated healthcare services, there could be greater emphasis on direct-to-patient health technology assessments, such as algorithm-based smartphone apps that assess the risk of skin cancer [76]. This technology, in combination with ever-improving telecommunication, could path the way for virtual consultations of the future.

### Adaptive Professional Environment

The COVID-19 pandemic has prompted reflection within the medical community regarding how the medical system functions, particularly in regards to the pressure and workload placed on staff. A thinly stretched healthcare system does not leave much room for sick leave, which is particularly problematic during a pandemic. Going forward, systemic changes, such as greater volume of staff with increased role-sharing, would alleviate some of the pressures medical staff face. A workplace with greater depth would be better positioned to adapt to significant adversity and demand, such as during a pandemic, with the hope that fewer services would be shut down during future events.

On a global level, the international scientific and medical communities must remember the importance of cooperation and collaboration. Working together (along with adequate funding) allowed for the fast development of COVID-19 vaccines, and these same processes can be used to overcome the significant setbacks in cancer care.

## Conclusion

To conclude, it is clear that if oncology is pushed too far to the sideline and disregarded, we are giving COVID-19 more power to spark future public health cancer crises, with an avalanche of late-stage diagnoses, unmet psychological need in oncology patients, further inequity in minority groups’ access to care and possibly maladaptive changes to practice. We must believe that ‘this experience made us better, stronger, and more human’ [47] in regards to the newfound scientific and technological advances and rejuvenated spirit of international collaboration. They are what have carried our medical system through the pandemic, and they must be enough to overcome the challenges ahead.
